# Cell therapies in ovarian cancer

**DOI:** 10.1177/17588359211008399

**Published:** 2021-04-22

**Authors:** Apostolos Sarivalasis, Matteo Morotti, Arthur Mulvey, Martina Imbimbo, George Coukos

**Affiliations:** Department of Oncology, Lausanne University Hospital, University of Lausanne, Lausanne, Switzerland; Department of Oncology, Lausanne University Hospital, University of Lausanne, Lausanne, Switzerland; Ludwig Institute for Cancer Research, Lausanne Branch, University of Lausanne, Lausanne, Switzerland; Department of Oncology, Lausanne University Hospital, University of Lausanne, Lausanne, Switzerland; Department of Oncology, Lausanne University Hospital, University of Lausanne, Lausanne, Switzerland; CHUV, Rue du Bugnon 46, Lausanne BH09-701, Switzerland

**Keywords:** adoptive cell therapy (ACT), cancer immunotherapy, neoantigens, ovarian cancer, tumor-infiltrating lymphocyte (TIL)

## Abstract

Epithelial ovarian cancer (EOC) is the most important cause of gynecological cancer-related mortality. Despite improvements in medical therapies, particularly with the incorporation of drugs targeting homologous recombination deficiency, EOC survival rates remain low. Adoptive cell therapy (ACT) is a personalized form of immunotherapy in which autologous lymphocytes are expanded, manipulated *ex vivo*, and re-infused into patients to mediate cancer rejection. This highly promising novel approach with curative potential encompasses multiple strategies, including the adoptive transfer of tumor-infiltrating lymphocytes, natural killer cells, or engineered immune components such as chimeric antigen receptor (CAR) constructs and engineered T-cell receptors. Technical advances in genomics and immuno-engineering have made possible neoantigen-based ACT strategies, as well as CAR-T cells with increased cell persistence and intratumoral trafficking, which have the potential to broaden the opportunity for patients with EOC. Furthermore, dendritic cell-based immunotherapies have been tested in patients with EOC with modest but encouraging results, while the combination of DC-based vaccination as a priming modality for other cancer therapies has shown encouraging results. In this manuscript, we provide a clinically oriented historical overview of various forms of cell therapies for the treatment of EOC, with an emphasis on T-cell therapy.

## Introduction

Epithelial ovarian cancer (EOC) is the most lethal gynecologic malignancy and the fifth deadliest cancer in women.^1^ In recent years, high-grade serous ovarian carcinoma (HGSOC), the most common histological subtype, has experienced many practice-changing treatment advances. These advances were mainly related to the incorporation of poly-[adenosine diphosphate (ADP)-ribose] polymerase (PARP) inhibitors into the treatment paradigm for both BRCA-mutant and for BRCA wild-type EOC tumors.^2^ Despite significant improvements in surgical and medical treatment, most EOC patients experience disease recurrence within 2–3 years, and successive progressions with shorter treatment-free periods, and eventually, death.^3^ Hence, there is an unmet need for novel and effective therapeutic options for patients with EOC.

Drug-based immunotherapy, such as immune-checkpoint blockade (ICB), has brought practice-changing advances in many hematological and solid tumors. Although EOC is considered a potentially immunoreactive tumor type, with the presence of intratumoral tumor-infiltrating lymphocytes (TILs) correlating with good clinical outcomes,^4,5^ the use of immunotherapy for EOC patients has failed to deliver clinically meaningful results so far.^6^ Indeed, despite durable responses in a subset of patients, many patients do not respond to the immunotherapies in current use.^7,8^

Advanced therapeutic medicinal products (ATMPs), such as adoptive cell therapy (ACT), represent an emerging potential treatment option for solid tumors, which could be of interest for EOC patients. This review aims to give a comprehensive overview of the previous developments and the current status of ACT in EOC.

## Adoptive cell therapies: an overview

ACT treatments are a type of personalized ATMP, manufactured specifically for each patient using their own cellular material (e.g. immune effector cells). This process utilizes autologous intra-tumoral or peripheral blood immune effector cells, which are extracted, expanded *ex vivo*, and often genetically modified, in an effort to enhance an anti-tumor response.^9^ The pioneering work at the National Institutes of Health (NIH) Surgery Branch to effectively grow immune cells *in vitro*, in particular, T cells, has paved the way for ACT in cancer immunotherapy.

ACT can broadly be classified into three different types: (a) expanded natural TILs; (b) T-cell receptor (TCR)-engineered T cells; and (c) chimeric antigen receptor (CAR)-modified T cells.^10^ All three of these cellular therapy types are currently being explored for EOC treatment, along with other strategies such as natural killer (NK) cells.

In one approach to ACT, which has been pioneered in patients with metastatic melanoma, TILs are cultured from resected cancer deposits, then expanded to large numbers *ex vivo*, and finally re-infused back into the patient.^11^ Alternatively, a similar treatment can be performed using peripheral blood-derived T lymphocytes (PBLs) that have been expanded following exposure to a select antigen *in vitro*.^12^ TIL therapy capitalizes on pre-existing spontaneous T-cell responses mediated by polyclonal populations of tumor-reactive T cells against mostly unknown tumor-specific antigens. Autologous T lymphocytes can also be genetically engineered to express a TCR that confers recognition of a specific tumor antigen.^13^ CAR-T cells are another application of an engineering approach that introduces a new synthetic receptor that redirects T cells to a cancer surface antigen. CARs combine an antigen recognition domain, typically a single chain variable fragment of a specific antibody, with an intracellular domain of the CD3-ζ chain or the FcγRI protein, to form a single chimeric protein.^14^ At present, all ACT approaches require conditioning of the host with pretreatment lymphodepleting chemotherapy (LTD), to overcome immunosuppressive mechanisms, such as regulatory T lymphocytes, and to create hematologic space to enable the ‘engraftment’ of the transferred lymphocytes. All these highly personalized treatments have the substantial advantage of being a potentially curative treatment if a durable anti-cancer immune response is generated.

## High-dose chemotherapy and autologous stem-cell transplantation (HDCT-ASCT)

The preconditioning LTD, apheresis, and re-infusion scheme used in ACT protocols are partially inspired by treatment protocols developed in 1980 in hematological stem-cell transplantation for the treatment of solid tumors. In this period, many solid tumors, including breast and EOC, were treated by high-dose chemotherapy (HDCT).^15,16^ This strategy was based on the observation that the cytotoxic effect of chemotherapy would increase proportionally with increased doses and eventually overcome tumor chemoresistance.^17^ The dose intensity of these protocols was five to ten times the usual standard and was leading to protracted hematological toxicity and bone marrow aplasia. Thus, HDCT required autologous stem-cell transplantation (ASCT) as a hematologic rescue.

Several phase I and II trials of HDCT using bone-marrow ASCT^18^ and one phase III trial have been carried out in EOC in the past.^19^ A survey of 421 patients who had undergone HDCT with ASCT in the US, identified 20 different chemotherapy regimens, usually a combination of agents such as melphalan, carboplatin, cyclophosphamide, and thiotepa. The 2-year progression-free survival (PFS) for this treatment was 12%, and the 2-year overall survival (OS) was 35%.^20^ The only randomized trial comparing sequential high *versus* standard-dose chemotherapy in first-line treatment of patients with EOC showed no statistically significant difference in PFS or OS.^19^ HDCT regimens were then progressively abandoned in favor of platinum doublets as the standard of EOC treatment.

Simultaneously, a less toxic regimen of ‘mini ablation’ developed for allogeneic bone-marrow transplantation showed immune-mediated graft-*versus*-leukemia effects.^21^ This raised the hope that a less toxic regimen could be adapted to a form of ‘transplantation,’ such as TIL therapy. The LTD regimen for ACT protocols was then refined until the current cyclophosphamide and fludarabine combination.^22^ Rather than a direct anti-tumor effect, this combination aims to suppress both the tumor microenvironment (TME) and the host’s immune system, thus optimizing engraftment and ACT product persistence.^23^

## Adoptive cell therapy with peripheral blood lymphocytes

The PBLs were amongst the first forms of ACT used for EOC treatment. In this technique, PBLs were isolated and expanded from peripheral blood mononuclear cell (PBMC) aphaeresis. The precursor CD8+ lymphocytes were then isolated and stimulated with specific tumor antigens to obtain a tumor-reactive ACT product. The ACT re-infusion was administered intravenously (i.v.) or intraperitoneally (i.p.).

Wright *et al*.^24^ conducted a pilot study using autologous PBMC-derived cytotoxic T-lymphocytes (CTL) stimulated *in vitro* with MUC1 peptide. Seven patients with recurrent EOC were planned to receive four cycles of i.p. CTL infusion. In anticipation of each cycle, a leukapheresis was performed. Although i.p. port complications prevented treatment continuation in 4/7 patients, the study showed this treatment’s feasibility. Interestingly one patient had a complete response (CR). The cytotoxicity-assessment revealed that despite an initial increase in CTL and immune parameters in the first month after infusion, CTL either decreased or plateaued from the second month.

The difficulties in obtaining high-affinity CTL in sufficient numbers prompted the investigation of *in vivo* priming approaches. Capitalizing on the restoration of immunity by dendritic cell (DC) anti-tumor vaccination,^25,26^ Kandalaft *et al*.^27^ reported the preliminary results of phase I sequential trial using whole-tumor antigen-pulsed dendritic cell (DC) vaccination followed by ACT using vaccine-primed PBL in heavily pretreated EOC patients. The treatment was feasible without relevant adverse events and generated anti-tumor immune responses and clinical benefit, including two patients with partial response (PR) and two patients with stable disease (SD) out of six patients.

## Adoptive cell therapy using tumor-infiltrating lymphocytes (TILs)

**Figure 1.**
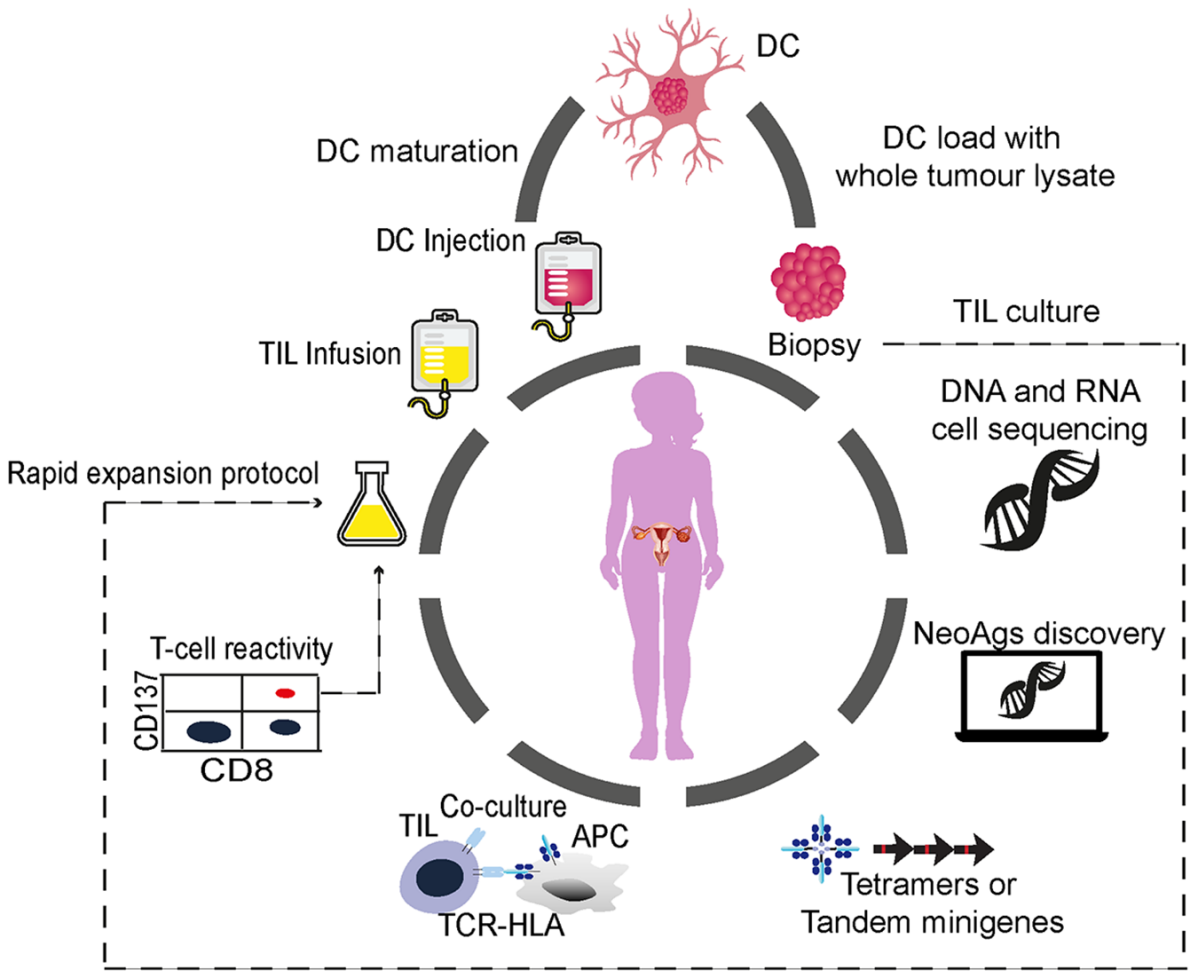
General schematic for using DC vaccines and TIL-ACT for patients with OC. A schematic is shown which describes the key steps in preparation of TIL-ACT for OC cancer therapy. The resected specimen and blood draw, generally *via* a leukapheresis procedure are taken from the patient. The resected specimen is divided into multiple tumor fragments that are individually grown in high-dose 6000 UI IL-2 for 7–14 days (pre-REP phase). For the ‘unselected’ TIL therapy (dashed line) the individual cultures are then moved to a rapid-expansion protocol (REP) in the presence of irradiated feeder lymphocytes, anti-CD3, and IL-2 before re-infusion into patients. The NeoAg-TIL therapy entails the sequencing of exomic or whole-genome DNA from tumor cells and healthy cells to call tumor-specific mutations. Corresponding minigenes or peptides encoding each mutated amino acid are synthesized and expressed in, or pulsed into, a patient’s autologous antigen-presenting cells (APCs) for presentation in the context of a patient’s HLA. The identification of individual mutations responsible for tumor recognition is possible with analysis of the T-cell activation marker, such as CD137 (CD8+ T cells), when they recognize their cognate target antigen. ACT, adoptive cell therapy; DC, dendritic cell; DNA, deoxyribonucleic acid; HLA, human leukocyte antigen; IL, interleukin; NeoAg, neoantigen; OC, ovarian cancer; RNA, ribonucleic acid; TCR, T-cell receptor; TIL, tumor-infiltrating lymphocyte.

ACT using TILs is based on the infusion of autologous CD4+ and CD8+ T lymphocytes expanded from tumors in the presence of high-dose interleukin-2 (IL-2) alone or in combination with other interleukins (Figure 1). TIL-ACT is based on the concept that the cellular immune response to cancer largely depends on conventional T cells, CD8+ and CD4+, that specifically target tumor-specific antigens such as cancer/testis antigens or cancer neoantigens (NeoAgs) that are derived from somatic genomic alterations that lead to the expression of immunogenic neo-epitopes.^28–31^

TIL-ACT has shown impressive results in metastatic melanoma and has reproducibly demonstrated having a curative potential with overall response rates of approximately 50%, including durable CR in up to 25% of treated patients.^32^

TIL-ACT has also been tested in EOC. However, the trials reporting data so far have shown a limited success of this strategy (Table 1).

**Table 1.** Clinical studies with TIL-ACT in EOC.

First author	Patient population	Patients (*n*)	Phase	Interventions/combinations
Aoki *et al*.^33^	Recurrent	17	Pilot	Cy (200 mg; day 2) +TIL (*n* = 7) or alternated cisplatin-based CT with TIL (*n* = 10)
Freedman *et al*.^34^	Advanced, platinum refractory	8/11	Pilot	IP TILs expanded in rIL-2 and low-dose rIL-2 IP
Ikarashi *et al*.^35^	Stage II–IV after PDS	12/22	I/II	TILs after cisplatin-based CT
Fujita *et al*.^36^	Stage II–IV, R0 after PDS and cisplatin-based chemotherapy	13/24	Pilot	Day 1: Cy (350 mg/m^2^), doxorubicin (40 mg/m^2^), cisplatin (50 mg/m^2^); 5-fluorouracil (350 mg/m^2^; from days 1–5), for 3–5 cycles; TIL infusion after CT
Freedman and Platsoucas^37^	Advanced platinum refractory	N/A	Pilot	N/A
Hua *et al*.^38^	Unknown	25	Double-blind RCT study	N/A
Freedman *et al*.^39^	Stage III or IV	2/22	Pilot	Patients received TIL (day 2) during a cycle that consisted of IP rIFN-g (days 1 and 4) and IP rIL-2 (days 2–5)
Pedersen *et al*.^40^	Progressive/recurrent	3/5	Pilot	60 mg/kg Cy for 2 days and 25 mg/m^2^ Flud for 5 days followed by TIL administration and decrescendo IL-2 (5 days)
Dudley *et al*.^41^	Recurrent platinum resistant	6	Pilot	Ipilimumab one dose (3 mg/kg) 2 weeks prior to tumor resection for TIL expansion; 2 days of Cy (60 mg/kg) followed by 5 days of Flud (25 mg/m^2^) before TIL infusion, nivolumab (3 mg/kg; q2w × 4) and low-dose o.d. IL-2 for 2 weeks.

ACT, adoptive cell therapy; CT, chemotherapy; Cy, cyclophosphamide; EOC, epithelial ovarian cancer; Flud, fludarabine; IL-2, interleukin-2; IP, intraperitoneal; N/A, not applicable; o.d., once daily; PDS, primary debulking surgery; q2w, twice per week; RCT, randomized controlled trial; rIFN-g; recombinant interferon-g; rIL-2, recombinant interleukin-2; TIL, tumor-infiltrating lymphocyte.

The first results of TIL-ACT in EOC were reported back in 1991 by Aoki *et al*.^33^ in Japan. In this trial, TIL-ACT was administered i.v. with or without cisplatin-based chemotherapy, after a single dose of cyclophosphamide 48 h before the TIL infusion. Although the suboptimal LTD doses compared with the current standard and the absence of adjuvant IL-2, seven cases of complete regression (with no recurrence for >15 months of follow up) and two cases of partial regression were reported.

Freedman *et al*.^34^ subsequently explored the use of i.p. TIL-ACT infusion in 11 recurrent EOC patients. All patients also received low-dose i.p IL-2. No LTD chemotherapy was used. Despite the interesting i.p. injection concept, there were no measurable responses, apart from some ascitic and blood CA125 level reduction in two and one patient, respectively. In 2000, the same group published a second attempt to treat EOC by i.p. TIL-ACT. In this trial, they used interferon gamma (IFN-γ) or IL-2 support after the infusion of the TIL product. Only 2/22 enrolled patients received i.p. TIL-ACT infusion due to the inability to expand TILs. No relevant results were thus available from this trial.^39^

In 1994, Ikarashi *et al*.^35^ compared the addition of TIL-ACT therapy after primary debulking surgery (PDS) followed by adjuvant chemotherapy in EOC patients. TILs were harvested from surgical debulking material, stimulated for 1 week with anti-CD3 followed by high-dose IL-2, and then frozen. At the end of cisplatin-based adjuvant chemotherapy, 10 patients received an i.v. infusion of thawed TILs. In this trial, all of the TIL-treated patients were alive at 26.5 months’ follow up. Unfortunately, no longer follow-up data were subsequently reported.

Fujita *et al*.^36^ subsequently reported the results of a phase I trial of TIL-ACT in the first-line setting. They enrolled 13 patients who had undergone PDS with no residual disease, followed by platinum-based adjuvant treatment. At the end of adjuvant therapy, the frozen TILs that were previously harvested and grown in IL-2 (100 UI/ml) and anti-CD3 were administered i.v. A control group of 11 patients who received standard first-line treatment without TIL-ACT was included for comparison. The 3-year disease-free survival rates were 82.1% for the TIL group compared with 54.5% for the control group.

More recently, Pedersen *et al*.^40^ reported the results of a pilot study where six patients with platinum-resistant EOC were treated with TIL-ACT after LTD chemotherapy, and subsequently stimulated by decreasing doses of post-infusion IL-2. TILs were harvested and grown with standard high-dose IL-2 (6000 IU) for the pre-REP (rapid-expansion protocol) phase followed with a REP phase of anti-CD3 and feeder cells.^42,43^ Despite that, TIL-ACT showed feasibility, with manageable toxicity; clinical outcomes were limited and mainly transient. SD was observed in four patients in the first 3 months, with two patients demonstrating durable SD at 5 months. Interestingly, infused TILs exhibited T-cell exhaustion markers (LAG3 and PD-1). Based on these results, the same group recently reported a cohort of six patients with late-stage EOC, which were treated with ipilimumab followed by surgery to obtain TILs and infusion of REP-TILs low-dose IL-2 and nivolumab.^41^ One patient achieved a PR, and five others experienced SD for up to 12 months. Furthermore, they showed that the addition of ipilimumab therapy improved the T-cell fold expansion during production and increased CD8+ T-cell tumor reactivity. Several clinical trials of TIL-ACT alone or in combination with ICB are ongoing in Europe [ClinicalTrials.gov identifiers: NCT04072263, NCT04611126, NCT03412526, NCT03992326] and in the US [ClinicalTrials.gov identifier: NCT03610490, NCT03318900].

Interestingly, the TIL-ACT trials in EOC dating from the 1990s have reported better outcomes than more recent trials (Table 1). Despite the difficulty in reproducing older datasets due to different LTD preparatory regimens, *in vitro* TIL growth culture methods, and IL-2 *in vivo* support,^44^ in these older TIL-ACT trials, patients were treated in the first-line setting compared with a late stage in the more recent ones. Indeed, earlier patient recruitment in cell-based therapies programs could theoretically facilitate the delivery of improved cellular products and minimize complicating co-morbidities associated with advancing metastatic cancer.^45^ It may also avoid the subclonal tumor heterogeneity developing after platinum-treatment pressure,^46^ making the isolation of TILs against clonal-NeoAgs more challenging.

### Neoantigen-based TIL-ACT in EOC

As highlighted in Table 1, none of the TIL-ACT trials in EOC to date have tested the TIL product for reactivity against tumor NeoAgs prior to infusion. NeoAg-specific T cells have been identified in several solid cancers, including EOC,^47,48^ and they have been identified as a major factor in the activity of T-cell responses both in patients treated with ICB^49,50^ and those treated with TIL-ACT.^30,51^

The NeoAgs discovery pipeline is an extensive process and is briefly summarized in Figure 1.^52,53^

In the context of EOC, the relatively low somatic point mutation load, high aneuploidy levels, and high levels of copy number alterations (CNAs) have been associated with low immunogenicity.^54,55^ However, as previously mentioned, a high density of intraepithelial CD8+ TILs is associated with an increased overall survival, as well as the presence of an immunoreactive gene expression signature.^4,55^ Moreover, these epithelial CD8+ TILs are negatively associated with tumor clonal diversity, reflecting immunological pruning of tumor clones by T cells with associated NeoAg depletion and loss of human leukocyte antigen (HLA)-I heterozygosity.^56^ This implies the existence of a close relationship between genotype, TME, and T-cell immune response in EOC,^57^ suggesting an important role of cytolytic T cells directed against cancer EOC NeoAgs during tumor evolution.

Intratumoral neoantigenome-directed T-cell responses have not been comprehensively evaluated in EOC until recently. In a pilot study on three patients with EOC, only one mutation (1/79, 0.01%) was detected to elicit a T-cell response.^47^ More recently, three other studies assessed the NeoAg landscape of EOC tumors. Deniger *et al*.^58^ studied a total of seven women with metastatic EOC, which were enrolled in a clinical trial evaluating autologous TIL-ACT therapy at the NIH. A total of 1714 putative NeoAgs were identified, with a median of 228 NeoAgs per patient. Only eight of these NeoAgs (0.03%, 8/228) were immunoreactive. As expected, all the immunoreactive mutations were unique to each patient. This paper’s most noteworthy results relate to TP53 missense mutations, the key initiating events in HGSOC. The authors identified mutation-specific TIL populations directed against two hotspot TP53 mutants (G245S and Y220C). Interestingly, both neo-epitopes, although distinct and non-overlapping, were presented in association with HLA-DRB3*02:02:010.

The same group subsequently reported the presence of intratumoral and PBL T-cell responses to defined TP53 hotspot mutations in a large cohort of patients with multiple tumor types.^59,60^ The idea of having TILs recognizing a key driver event in EOC, as well as in other tumors, is indeed fascinating. However, it is evident through the lack of cancer-cell elimination and tumor immune escape, that mechanisms capable of preventing T-cell-mediated death against driver mutations might be an extremely early event in cancer evolution. In fact, the presence of widespread driver mutations in healthy tissue, such as pathogenic TP53 mutations in normal endometrium epithelium,^61^ may promote immune tolerance early in life against these potential neo-epitopes and thus hampering the possibility as immunogenic targets.

Bobisse *et al*.^62^ evaluated the neo-epitope landscape in TILs and PBL of 19 immunotherapy-naïve patients with recurrent advanced EOC. A total of 776 candidate neo-epitopes were predicted *in silico* to bind with high affinity to patients’ cognate HLA-I alleles, with a range of 1–133 neo-epitopes predicted per patient. In total, PBLs or TILs recognizing neo-epitopes were identified in two thirds (9/14) of the patients, for a total of 14 distinct neo-epitopes (0.02%, 14/776). Neo-epitope recognition was largely discordant between PBLs and TILs, and the latter also exhibited markedly higher functional avidity against neo-epitopes compared to PBLs. Westergaard *et al*.^63^ successfully established and expanded TILs from 34 tumor specimens of EOC and demonstrated autologous tumor-cell recognition in >50% of the patients. They were also able to identify two immunogenic neopeptides derived from a patient-derived EOC cell line with LC-MS-based immunopeptidomics. Furthermore, NeoAg-specific TILs were isolated and further expanded *in vitro.*

All three studies reached important conclusions. First, TILs recognizing tumor NeoAgs can be specifically identified in a large proportion of EOC patients with different techniques, even if such tumor type harbors a relatively low mutational load. Conversely, the rate of NeoAg immunogenicity filtered by the current bioinformatics pipeline is very low (<0.1%) and similar/lower to that seen in other studies,^64,65^ despite cross-validation of NeoAg prediction being very low at the moment.^66^

In conclusion, TIL-ACT has been shown as feasible, but with modest results, in treating patients with EOC. The lack of TIL-ACT efficacy in this setting may result from several factors such as an inefficient expansion of TILs *ex vivo*, suboptimal LTD conditioning, or IL-2 support in older studies.^67^ Recent studies have shown that neo-epitope-specific clones can be isolated from EOC patients extending opportunities for mutanome-based personalized immunotherapies in this tumor type.

## CAR-T and TCR-based therapy in EOC

TCR and CAR-T therapy are two types of ACT that genetically modify T cells to treat cancers. In both these ACT procedures, naïve T cells are isolated by aphaeresis of peripheral blood, but they differ in their antigen recognition machinery.^68^

TCRs employ a glycoprotein alpha–beta chain heterodimer that recognizes peptides presented on the cell surface by HLA molecules. CARs, on the other hand, are engineered to display antigen-binding fragments of a specific antibody fused to intracellular T-cell signaling domains. Thus, the bioengineered TCR can elicit an HLA-restricted immune response, while the CAR-T cells can recognize tumor-cell-surface antigens and elicit an immune response independently of HLA-antigen recognition.^69,70^ Moreover, the TCRs confer recognition to epitopes derived from proteins residing within any subcellular compartment while CAR-T cells can only recognize cell-surface antigens.

Much of the work on CAR-T and TCR therapy in EOC has been directed at tumor-associated antigens (TAAs), which are self-proteins abnormally expressed by cancer cells. These shared antigens can be separated into three main groups: the overexpressed antigens, which are normal surface proteins expressed in elevated levels on cancer cells, but in lower levels in normal cells (e.g. hTERT, HER2, mesothelin, and MUC-1); tissue-specific TAAs, which are shared between tumors and the normal tissue of their origin [e.g. tyrosinase, gp100, MART-1, prostate-specific antigen (PSA)]; and TAAs whose expression is normally restricted to male germline cells [i.e. cancer testis (CT) antigens, such as MAGE-A1, MAGE-A3, and NY-ESO-1].^71^

Some of the EOC antigens investigated *in vitro* and *in vivo* for CAR-T development are FRβ, mesothelin, MUC-1, HER2, and folate-receptor alpha (FRα).^72^ The first phase I trial of CAR-T in EOC used FRα as target and a first-generation CAR design, that is, comprising a CD3ζ endodomain but lacking a costimulatory domain. While this study demonstrated CAR-T’s safety in six patients with recurrent EOC, the results were disappointing, with no clinically evident tumor responses, most likely due to low expression of the transgenic CAR and poor persistence of the transferred T cells.^73^ The persistence of engineered T cells can be dramatically improved by using a human single-chain variable fragment and by adding costimulatory signaling domains to the CAR. A phase I trial with the third-generation CAR-T design is currently recruiting [ClinicalTrials.gov identifier: NCT03585764].

CAR-T against mesothelin (CART-meso), a consistently overexpressed protein carrying a negative prognostic role in EOC, was tested in a phase I/II trial to treat metastatic cancer patients, including EOC. Unfortunately, the trial was terminated due to slow/insufficient accrual [ClinicalTrials.gov identifier: NCT01583686]. Preliminary results of second-generation CART-meso are encouraging,^74^ although final data are not available yet.

More recently, Haas *et al*.^75^ used lentiviral-transduced CART-meso in 15 advanced solid tumors, including EOC patients. The treatment was well tolerated but showed limited efficacy with SD as the best overall result. Novel interesting strategies using mesothelin CAR and a suicide strategy that induces apoptosis in cancer cells after binding *via* iCaspase9 release are ongoing in patients with pleural tumors [ClinicalTrials.gov identifiers: NCT02414269 and NCT02792114] and might represent an interesting strategy in EOC.

Other targets, such as MUC16 (CA125), were tested in a phase I trial using an i.v. and i.p. CAR-T modified to secrete IL-12. Although no significant toxicity was seen, the best observed clinical response was SD in a cohort of 18 heavily pretreated EOC.^76^ A vast range of clinical trials using third-generation CAR-T against known EOC TAAs, such as the one cited earlier, are ongoing and have been recently reviewed.^77^

Similarly, HLA-A2-restricted TCRs specific for epitopes from known EOC antigens such as NY-ESO-1, WT1, p53, MAGE-A4 are available for clinical application.^44,78^ In particular, NY-ESO-1, a CT antigen, over-expressed in more than 40% of EOC patients,^79,80^ has been tested in several phase I/II clinical trials, with TCR-ACT showing clinical benefits. Several other trials against NY-ESO-1 are currently ongoing in EOC patients [ClinicalTrials.gov identifiers: NCT01567891, NCT03691376, NCT02457650, NCT02869217, NCT03159585]

In contrast to CAR-T cells’ striking successes in treating patients with hematological malignancies, no equivalent achievements have been demonstrated to date in patients with solid tumors, including EOC. The limited number of targetable membrane antigens and their heterogeneous expression, difficulties in T-cell fitness and survival before reaching the tumor sites, and impaired trafficking to tumor sites have proven key obstacles for the success of CAR-T and TCR-based therapies in solid tumors and EOC. Technological advances in immune-engineering with viral vectors or for gene-editing with CRISPR–Cas9 technology^81^ might open important avenues to tackle some of these key barriers to ACT, such as overcoming T-cell inhibitory signal or T-cell immunosuppressive metabolic insults^82,83^ from the TME. An ongoing clinical trial, which includes patients with EOC, is testing the effect of CRISPR-Cas9mediated PD-1 knocked-out CAR-T cells against mesothelin [ClinicalTrials.gov identifier: NCT03747965], and results are awaited.

The problem of antigen heterogeneity in solid tumors has led to the attempt of strategies that target multiple TAAs simultaneously. The use of aFRα bispecific T-cell engager has been shown to increase CAR-T cells’ efficacy in preclinical models of ovarian, colon, or pancreatic cancer.^84^ Other examples include avidin-linked CARs combined with biotinylated antibodies^85^ or fluorescein isothiocyanate fluorophore conjugated to TAA-binding molecules,^86^ in an effort to successfully target heterogeneous solid tumors such as EOC.

## Natural killer cells (NK)-based immunotherapy

Natural killer (NK) cells are another type of immune cell that can kill target cells through similar cytotoxic mechanisms as CD8+ cells. There has been a renewed interest in the use of NK cells as a source for genetically modified immune cell-based immunotherapy (CAR-NK) due to the reduced risk of off-target toxicity compared with CAR-T cells, the possibility of both CAR-dependent and NK cell receptor-dependent mechanisms of tumor killing, and their reduced risk of alloreactivity and thus graft *versus* host disease even with allogeneic NK cells.^87^ In fact, since NK cells do not require HLA matching to a specific patient, it is feasible, and safe to transfer cells across allogeneic barriers, thus opening the possibility of transferring off-the-shelf NK cells. Moreover, cytokine-release syndrome (CRS) and neurotoxicity are less likely to occur in CAR-NK immunotherapy partly due to a different spectrum of the secreted cytokines: activated NK cells usually produce IFN-γ and GM-CSF, whereas CAR-T cells predominantly induce cytokines, such as IL-1a, IL-2, IL-6, and tumor necrosis factor alpha that are positively associated with CRS and severe neurotoxicity.^87,88^

NK-based immunotherapy strategies have been studied for a long time in treating EOC,^89^ also based on the *in vitro* results showing that allogeneic NK cells exhibit anti-tumoral activity against OC cells isolated from ascites.^90^ Early attempts at inducing NK cells for anti-cancer function included priming of lymphokine-activated killer (LAK) and cytokine-induced killer (CIK) cells. LAK and CIK cells originate from naïve lymphocytes, which are ‘activated’ or ‘induced’ by IL-2 alone (LAK) or following IFN-γ stimulation *ex vivo* (CIK).^91^ In clinical trials, LAK cells exhibited limited clinical response and high peritoneal fibrosis rates.^92,93^ Instead, CIK cells, which are characterized by the expression of a CD3+CD56+ cell phenotype and functional properties of NK cells, were used in an EOC phase III study of ACT following PDS and carboplatin/paclitaxel chemotherapy.^94^ The results showed an improvement of PFS in the experimental arm compared with the control group (37.7 months *versus* 22.2 months, respectively).

Geller *et al*.’s^95^ phase II study used allogeneic, haploidentical donor NK cells in combination with chemotherapy to treat patients with recurrent EOC. NK i.v. infusion with IL-2 stimulation was tested in 14 heavily pretreated recurrent EOC patients. Overall, the treatment was well tolerated apart from one grade 5 event due to tumor lysis syndrome. Interestingly, 4/14 patients experienced PR, and 8/14, SD. In 2016, Yang *et al*.^96^ reported allogeneic NK cell ACT from an early basket trial. Two EOC patients were included. No LTD chemotherapy regimen was used. Though an anti-tumor effect of the adoptively transferred NK cells could be observed with 8/17 patients showing SD, their persistence *in vivo* was shorter (between 1 and 4 days) in comparison with other clinical trials.

NK-ACT is also under evaluation for i.p. administration in the APOLLO phase I study [ClinicalTrials.gov identifier: NCT03213964]. Preliminary results presented, but not yet published, with the i.p. delivery of FATE-NK100 cells, show safety, and a clinical benefit in 3/9 patients treated. A summary of all clinical trials employing NK and NK-related cells for the treatment of EOC is presented in Table 2.

**Table 2.** Clinical studies with NK-cell based immunotherapies in OC.

NK cell intervention	Phase, date, (status)	OC study population (*n*)	Primary outcomes	Results	Reference/ClinicalTrials.gov identifier
Allogeneic NK cells (with IL-2)	Phase II, 2008–2010 (terminated due to toxicity)	12	To evaluate the *in vivo* expansion of NK cell product	PR (3), SD (8), PD (1)	NCT00652899
Allogeneic NK cells (with IL-2)	Phase II, 2010–2014 (completed)	13 patients with HGSOC and breast cancer	Response rate by RECIST at 3 months	N/A	NCT01105650
Cord blood cytokine induced killer cells	Phase I, 2012–2014 (completed)	4	Response rate by RECIST	2 PR and 2 SD	Zhang *et al*.^97^
Radiofrequency ablation and cytokine-induced killer cells	Phase II, 2015–2016 (active, not recruiting)	50	RFS (timeframe: 1 year)	N/A	NCT02487693
NK cells with cryosurgery	Phase I/II, 2016	30	Response rate by RECIST	N/A	NCT02849353
FATE-NK 100 (CMV+ donor NK cells with IL-2)	Phase I, 2017–2025 (recruiting)	10	Maximum tolerated dose of FATE-NK100 (timeframe: 1 year)	N/A	NCT03213964
NKG2D-ligand targeted CAR-NK	Phase I, 2018–2019 (recruiting)	30 (including OC)	Occurrence of AEs	N/A	NCT03415100
Primary NK cells	Phase I/II, 2018–2022 (recruiting)	200 solid cancer including OC	Incidence of toxicity induced by NK infusion (timeframe: 6 months)	N/A	NCT03634501
6B11-OCIK	Phase I, 2018 (recruiting)	10 Recurrent platinum resistant	3-year PFS	N/A	NCT03542669
IP Infusion of *ex-vivo*-cultured allogeneic NK cells	Phase I, 2018 (recruiting)	12 recurrent HGSOC	6-month AE rates	N/A	NCT03539406
Anti-mesothelin CAR-NK	Phase I, 2018 (not yet recruiting)	30	Occurrence of AEs	N/A	NCT03692637

AE, adverse event; CAR, chimeric antigen receptor; CMV, cytomegalovirus; HGSOC, high-grade serous ovarian carcinoma; IL, interleukin; N/A, not available; NK, natural killer; OC, ovarian cancer; PD, progressive disease; PFS, progression-free survival; PR, partial response; RFS, recurrence-free survival; SD, stable disease.

As of January 2021, 17 ongoing CAR-NK cells studies were registered in the ClinicalTrials.gov, of which seven were targeting metastatic solid tumors expressing TAAs. In particular, a phase I trial is evaluating the role of anti-mesothelin NK-CAR cells in patients with EOC expressing >50% of mesothelin after standard chemotherapy [ClinicalTrials.gov identifier: NCT03692637]. An NK-CAR targeting NKG2DL (a molecule commonly upregulated in solid tumors) is being evaluated in solid tumors, including EOC [ClinicalTrials.gov identifier: NCT03415000].

## Dendritic-cell vaccination

The application of vaccination for cancer treatment relies on the capability of vaccines to train the immune system. Through vaccine-training the host immune system can accurately detect and kill a variety of offending noxae, including cancer. This strategy was broadly tested in the field of infectious diseases. Cancer vaccines can be classified into different categories: (a) cell-based vaccines; (b) peptide/protein; (c) epigenetic; and (d) genetic vaccines, according to the method of choice to deliver the selected antigens.^98^

So far, around 50 trials with vaccination approaches have been performed in patients with EOC with no statistically clinical differences detected by vaccine type or treatment schema.^99,100^ Some of these vaccines have targeted a single antigen such as CA125, MUC1, Her2, p53, and NY-ESO-1. In contrast, others used multiple peptides from various antigens or a whole-tumor antigen approach created with tumor cells, autologous tumor lysate, or tumor-derived ribonucleic acid.^101–103^ Overall, most trials demonstrated low therapeutic effects with a median PFS of 13.0 months, mainly due to the low expression of the target antigen, tumor heterogeneity, and the patient population.^100,104^

A prolongation of PFS and OS in recurrent EOC was demonstrated in several clinical trials investigating the efficacy of NY-ESO-1-based vaccines,^105,106^ dendritic cell (DC) vaccines based on a pool of peptides (Her2/neu, hTERT, and PADRE peptides),^107^ or whole-tumor vaccines of autologous tumor cells infected with Newcastle disease virus,^108^ or viral oncolysate vaccine generated from EOC cell lines infected with influenza-A virus.^109^

DC vaccination with whole-tumor lysate might be a relevant strategy for EOC treatment, as surgery is a crucial component of EOC treatment, and therefore sufficient tumoral material should be available (Figure 1). Advantages of DC vaccination with autologous tumors and lysates include the agnostic targeting of the full repertoire of patient-specific TAAs and NeoAgs. This can stimulate a broad polyclonal T-cell response with priming of CD4+ and CD8+ T cells independent from the patient’s HLA haplotype.^110,111^ The pretreatment of EOC cells with hypochlorous acid (HOCl) oxidation (OCDC vaccine)^112^ has been shown to increase the overall vaccine immunogenicity.^113,114^ The intranodal injection of autologous DCs pulsed with the OCDC vaccine was evaluated in a pilot study of five subjects with recurrent EOC.^114^ Subsequently, other groups confirmed the safety of DC vaccination in EOC.^115,116^

We previously reported a two-step pilot clinical study testing the feasibility, safety, and clinical outcomes of DC vaccination using autologous tumor-cell-lysate supernatants in combination with bevacizumab and oral metronomic cyclophosphamide in recurrent OC.^27^ Subjects who achieved at least SD after the vaccine but failed to exhibit a complete remission were enrolled in a second trial, involving LTD and ACT of autologous vaccine-primed CD3/CD28-costimulated T cells. Despite the relative low frequency of tumor-specific T cells elicited by this vaccine (<1 tumor-reactive T cell per 500 PBLs), an overall clinical benefit of 50% was seen in this heavily pretreated population. This strategy demonstrated T-cell responses against known EOC antigens and, more importantly, a close correlation between clinical benefit and immune response.

More recently, we reported further results of a pilot clinical trial testing a personalized vaccine generated by autologous DCs pulsed with OCDCs.^26^ The vaccine was injected intranodally in platinum-treated, immunotherapy-naïve recurrent EOC patients. OCDC was administered alone (cohort 1, *n* = 5), in combination with bevacizumab (cohort 2, *n* = 10), or bevacizumab plus low-dose intravenous cyclophosphamide (cohort 3, *n* = 10) until disease progression or vaccine exhaustion. Vaccination induced T-cell responses to autologous tumor antigen and significantly increased survival in this heavily pretreated population, with an improvement in median PFS to 11.1 months from 4.1 months in the historical control population. In particular, longer PFS was seen in patients who produced tumor-reactive T cells after vaccination. The 2-year OS rate of the responder patients was 100%, whereas the 2-year OS of non-responders was 25%. Importantly, DC vaccination amplified the T-cell response to neo-epitopes with the emergence of novel T-cell clones of markedly higher avidity against previously recognized neo-epitopes.

This is particularly important, as the OCDC vaccine could be integrated as a priming modality before other immunotherapeutic strategies. The consequent anti-tumoral post-vaccination T-cell responses could be interrogated for the subsequent design of synthetic vaccines or T-cell therapies against newly recognized TAAs and/or tumor neo-epitopes. This combination of DC vaccines and TIL therapy has shown encouraging results in patients with melanoma.^117^ Moreover, OCDC may prime patients, especially those lacking TILs, by inducing antitumor immunity, which could benefit from ICB treatment.

Similar to what is seen for T-cell therapy, the ability to identify NeoAgs through tumor deoxyribonucleic acid (DNA)-genomic sequencing has shifted the focus to investigate the clinical feasibility of personalized recombinant NeoAgs vaccines.^118,119^ For this reason, a phase I/II, randomized two-cohort, single-center study was developed to compare the immunogenicity and assess the safety of a personalized peptide (with a pool of 10 prioritized NeoAg-based peptides), pulsed DC vaccine (PEP-DC) alone, or OCDC vaccine followed by PEP-DC (PEP-DC2), in combination with low-dose cyclophosphamide.^120^

Despite the reported limited clinical efficacy of DC-based therapeutic vaccination, this treatment modality has been associated with clinical activity in a subset of EOC patients representing a promising therapeutic option, alone or in combination, with other immunotherapies. Further studies are needed to define the best patient-candidate characteristics for therapeutic vaccine treatment, along with the optimal selection of the route of administration and neo-antigen selection.

## Challenges of cell therapy in ovarian cancer

Despite targeting various antigens (Ags) and combining ACT with chemotherapy and anti-angiogenic agents, the exciting results of cell therapies in hematological malignancies and melanoma have not yet been reproduced in EOC, showing so far limited anti-tumor activity in early-phase clinical trials.^121^

An ‘ideal’ target antigen should be highly and homogeneously expressed throughout the tumor, across multiple patients, and have minimal to no expression in normal tissues. Unfortunately, these characteristics do not apply to most currently explored targets for either CAR-T or TCR-T therapies in EOC. Current targets are abhorrently expressed antigens such as TAAs, which may be present at low levels in normal tissues; they might have heterogeneous Ag expression and have undergone thymic tolerance.^122^ Therefore, research has focused on targeting cancer stem cells or tumor-initiating cells. A peculiarity of EOC, however, compared with other types of tumors, is its unknown putative site and cell of origin.^123^ This might still preclude the identification of lineage markers as seen in hematological malignancies.

NeoAgs derived from non-synonymous mutations are instead tumor specific; they have not undergone central tolerance, and thus, are ideal candidates to exploit for cell therapy. However, a genomic peculiarity of HGSOC is its relatively low somatic point mutation load, high aneuploidy levels, and high levels of CNAs.^55^ These features have been associated with low immunogenicity due to the low presence of NeoAgs. Moreover, the majority of EOCs are diagnosed at an advanced stage, FIGO III–IV with multiple metastatic sites that are heterogeneous in terms of genomics and T-cell infiltration patterns.^56^ Thus, identifying clonal NeoAgs, potentially capable of simultaneously targeting multiple lesions, is a challenging task that might require access to various biopsies, which might not be clinically manageable in late-setting diseases.

Although the identification of neo-epitope-specific CD8+ T cells has been reported in ~90% of patients evaluated,^62^ these are patient specific, limiting this approach’s broad application. Moreover, most epitopes showing significant immune response in solid cancer are derived from intracellular proteins that are difficult to target with standard CAR-T cells.^124^

Another important barrier for the success of cell therapies in solid tumors is the capacity of antitumor effector immune cells to infiltrate and persist at the tumor site.

The EOC TME is intrinsically heterogeneous among both patients and tumors. Furthermore, beyond tumor heterogeneity, it has been reported that both immune-cell-excluded and inflammatory phenotype microenvironments may exist within the same tumor.^57,125^ This is an important challenge for the successful application of therapies that target the TME, such as ICB, and therapies requiring a permissive TME to act, such as cell-therapies. Moreover, a huge plethora of biochemical pathways and molecules present in the TME, and ascitic fluid in patients with EOC has been linked to immunosuppressive phenotypes which can lead to T-cell anergy and desertification.^54,126^ It appears evident that a better understanding of the key mechanisms leading to inflammation/desertification, as well as leading to T-cell exhaustion/anergy in the EOC TME, will be important to rationally design genome-engineered strategies and combination therapies to overcome this obstacle.

In particular, the choice of the right Ags and the improvement of T-cell characteristics for enhanced anti-tumor activity despite immunosuppressive TME are key factors for the further success of EOC cell therapies.

## Conclusions

Despite high rates of response to initial treatment, EOC has a high recurrence rate and has yet to show a significant response to available immunotherapeutic agents. Cell therapies have transformed the treatment paradigm for patients with hematologic malignancies; however, the translation of this success to the unmet need of EOC patients is ongoing. Cell-based therapies in EOC have been explored in early-phase studies from a few highly specialized centers, with modest clinical results, raising concerns for the future development of these potentially curative therapeutic approaches.

Previous experience with ACT-TIL has shown the feasibility of this complex approach in the setting with EOC. The incorporation of new technologies such as DNA sequencing, DNA synthesis, and genetic screening tools to identify individual patient-specific NeoAgs with high sensitivity, specificity, and at scale, might broaden the application of this type of treatment to a broader group of EOC patients in coming years. Vaccination strategies have, to date, mainly encompassed shared TAAs and have been met with limited success.

A better understanding of the T-cell biology (T-cell exhaustion) driven by the peculiar EOC TME will be a crucial step in developing ACT for these patients. It will drive advances in T-cell engineering and clinical trial design with combination therapies. The immunosuppressive TME needs to be tackled to improve CAR-T and TIL-ACT products’ activity and persistence, and immune-engineering innovative solutions are currently being developed. Similarly, as T-cell engineering technology is streamlined, it might gain a prominent role in combination with other strategies. We believe that a better understanding of these key biological questions, along with technological developments will be key to broadening the use of cell therapeutics and ultimately improve EOC patients’ clinical outcomes.
