# Corrigendum to “Cell therapies in ovarian cancer”

**DOI:** 10.1177/17588359211028457

**Published:** 2021-06-17

Sarivalasis, A, Morotti, M, Mulvey, A, *et al.* Cell therapies in ovarian cancer. *Ther Adv Med Oncol* 2021; 13: 1–17. DOI: 10.1177/17588359211008399

The Conflict of Interest statement was incorrect in the published version of the article. The correct version is given below. The online version of the article has been corrected.

Dr. Coukos has received grants from Celgene, Boehringer-Ingelheim, Roche, BMS, Iovance Therapeutics, Kite Pharma. The institution Dr. Coukos is affiliated with has received fees for Dr Coukos’ participation in advisory board or for presentation at a company sponsored symposium from Genentech, Roche, BMS, AstraZeneca, NextCure, Geneos Tx and Sanofi/Avensis. Dr. Coukos has patents in the domain of antibodies and vaccines targeting the tumor vasculature as well as technologies related to T-cell expansion and engineering for T-cell therapy. Dr Coukos holds patents around antibodies and receives royalties from the University of Pennsylvania regarding technology licensed to Novartis.

Dr Sarivalasis has received research grants from Roche. The institution Dr Sarivalasis is affiliated with has received consultancy and advisory fees from Roche, Novartis, GSK, Tesaro, BMS, Celgene, BMS, AstraZeneca. The institution Dr Sarivalasis is affiliated with has received travel fees from Roche, GSK, Tesaro, Clovis, AstraZeneca, MSD, Pfizer, Amgen, Celgene, Novartis.

